# Sex-Specific Associations in Nutrition and Activity-Related Risk Factors for Chronic Disease: Australian Evidence from Childhood to Emerging Adulthood

**DOI:** 10.3390/ijerph15020214

**Published:** 2018-01-26

**Authors:** Erin Hoare, Sarah R. Dash, Garry L. Jennings, Bronwyn A. Kingwell

**Affiliations:** 1 Metabolic and Vascular Physiology, Baker Heart and Diabetes Institute, Melbourne, VIC 3004, Australia; Sarah.Dash@baker.edu.au (S.R.D.); garry.jennings@sydney.edu.au (G.L.J.); Bronwyn.Kingwell@baker.edu.au (B.A.K.); 2 Food and Mood Centre, Centre for Innovation in Mental and Physical Health and Clinical Treatment, School of Medicine, Faculty of Health, Deakin University, Melbourne, VIC 3004, Australia; 3 Sydney Medical School, University of Sydney, Sydney, NSW 2006, Australia

**Keywords:** cardiovascular disease, diabetes, sex differences, lifestyle risk factors, nutrition, physical activity, obesity, early life

## Abstract

Global assessments of burden of disease suggests there are sex differences in risk factors for chronic disease, including overweight/obesity, dietary patterns and habitual physical activity. Given that prevention efforts aim to target such factors to reduce disease risk, the age at which sex differences may occur is of particular interest. Early life to young adulthood is the optimal time for intervention, with lifestyle habits typically forming during this period. This study aimed to identify the sex differences in risk factors for chronic disease during childhood (5–9 years), adolescence (10–17 years) and emerging adulthood (18–25 years) in a large population-representative Australian sample. Among children in this study (*n* = 739), no sex-related differences were observed. Among adolescents (*n* = 1304), females were more likely than males to meet daily fruit and vegetable recommendations (12.9% vs. 7.5%; OR = 1.84, 95% CI = 1.16, 2.93, *p* < 0.05). Among emerging adults (*n* = 909), females were less likely to be overweight/obese (30.1% vs. 39.8%; OR = 0.65, 95% CI = 0.44, 0.95, *p* < 0.05) and more likely to meet physical activity recommendations (52.1% vs. 42.3%; OR = 1.44, 95% CI = 1.01, 2.06, *p* < 0.05). These findings suggest that sex differences for risk factors of chronic disease occur during adolescence and emerging adulthood, although the differences are not consistent across age periods. From adolescence onwards, it appears that females exhibit lower risk factors than males and a life span approach to risk factor monitoring is warranted.

## 1. Introduction

Modifiable lifestyle risk factors, including physical inactivity, poor diet, alcohol consumption and smoking, have driven global increases in non-communicable chronic diseases. Lifestyle risk factors also underpin clinical risk factors such as high blood pressure, dyslipidaemia and overweight/obesity, and the surveillance of clinical and lifestyle factors now forms a major component of disease management and prevention efforts.

Critically, recent evidence suggests that many risk factors are sex-specific. For example, the Global Burden of Disease Study 2016 [1] revealed that the three leading risk factors attributable to disability-adjusted life years (DALYs) for males were smoking, high systolic blood pressure, and low birthweight [1], whereas the leading risk factors for women were high systolic blood pressure, high body mass index and high fasting plasma glucose. This global study assessed adult populations, however, whether these differences appear during early life to young adulthood, when habitual lifestyle behaviours develop, is of interest. It is during this age period when prevention interventions are often implemented, aiming to form lifestyle habits that carry on into older adulthood. While sex-specific trends have been observed among adults at a global level, it remains unclear whether such differences are observed across socioeconomic settings. Lifestyle drives much of the health burden of the population despite significant medical and scientific advancements [2]. 

Life expectancy in Australia has grown steadily over time but with the ageing population, lifestyle and social changes, chronic diseases such as diabetes, cardiovascular disease and cancer have increased in absolute terms [2]. In 2014–2015 there were approximately 1.2 million individuals living with diabetes (5.1%), 1.2 million living with heart disease (5.2% of the population), and 370,100 living with cancer (1.6%) [3]. With regard to the underlying risk factors, approximately 63% of Australian adults were overweight or obese, 5% met daily fruit and vegetable recommendations of two servings of fruit and five servings of vegetables, and just over half (56%) met physical activity guidelines of 150 min per week [3]. Sex differences occur within these epidemiological trends. For example, diabetes and cardiovascular diseases were more prevalent among Australian men compared to women, and cancer affected a greater proportion of men than women at older ages (75+ years). Men aged 45 years and over were more likely to be overweight or obese than females in the same age group (79% vs. 66%). Females were more likely to meet fruit and vegetable guidelines than males and physical activity levels were similar for males and females. Additionally, health outcomes had disproportionate negative effects for individuals experiencing socio-economic disadvantage, and any attempt to examine health status must account for such health inequalities [2]. 

Early life is an important window of opportunity, given that individual risk and protective behaviours are often developed and refined during this age period, and are likely to contribute to health outcomes later in adulthood. For example, a review investigating age-related consequences of obesity found that childhood obesity is associated with metabolic syndrome, hypertension, and type 2 diabetes in adulthood as well as obesity itself and related complications [4]. Physical activity during adolescence reduces the risk of cardiovascular disease and associated risk factors in adulthood [5,6]. The same protective effects have been observed for healthful diets during adolescence, and cardiovascular disease risk in adulthood [7]. The transitional life phase of emerging adulthood (18–25 years) is a recent area of interest in health prevention, due to significant life events occurring during this period (e.g., commencing tertiary studies/full time employment, living independently) [8]. Nationally-representative studies have reported significant weight gain during emerging adulthood that is greater than observed during other age periods [9,10,11]. Physical activity often declines during emerging adulthood, and a decline in overall-diet quality (e.g., increased fast-food and sugar-sweetened beverage (SSB) consumption) has also been reported [12,13]. Although highly complex and ultimately reflecting a gradual transition as opposed to distinct age categories, these age periods hold significant health challenges for individuals and communities and are of great public health interest.

Due to the need for surveillance of risk and protective factors across the life span, the aim of this study is to examine key nutrition, physical activity and other health characteristics among Australian children (5–9 years), adolescents (10–17 years) and emerging adults (18–25 years) and to investigate differences in risk and protective factors between males and females, identifying during which age period differences occur. These associations are examined in the context of socio-economic status, given the known impact of advantage and disadvantage on health outcomes. 

## 2. Materials and Methods 

### 2.1. Study Design

The 2011–2012 Australian Health Survey (2011–2012 AHS) was a comprehensive, Australia-wide survey conducted by the Australian Bureau of Statistics to assess the health, risk factors, health service and medication usage of urban and rural-dwelling Australians [14]. The details of survey design and data collection provided here are further summarized elsewhere [15]. The 2011–2012 AHS used a stratified, multistage area sample of private dwellings covering approximately 97% of Australia. The National Nutrition and Physical Activity Survey (NNPAS) formed one component of the suite of surveys from the 2011–2012 AHS which included information on physical activity, sedentary behaviour and dietary intake [16]. The NNPAS was conducted with a random sub-sample of residents within selected dwellings with one adult and, where applicable, one child aged 2–17 years. Trained ABS interviewers conducted personal interviews in sample dwellings with selected residents over 18 years. One adult nominated by the household was interviewed about a child 2 years and over, and individuals aged 15–17 years could be personally interviewed with parental consent. Approximately 15,565 households adequately responded to the AHS; 9519 households adequately responded to the NNPAS. When combined, NHS and NNPAS provide a core sample of 25,080 households and 31,837 respondents aged 2 and over. In the current study, participants aged 5–25 years inclusive with complete available demographic, dietary, and physical activity data were included, allowing for a total sample of 2952. Further information relating to survey design, data collection and response rates can be found elsewhere [15].

### 2.2. Measures

#### 2.2.1. Demographics

Age in single years and biological sex were self-reported. The Socio-Economic Index for Areas (SEIFA) was derived from the area in which the survey respondent lived, to determine level of socio-economic advantage/disadvantage (lowest 20% = most disadvantaged, highest 20% = least disadvantaged) [17].

#### 2.2.2. Risk and Protective Factors

The risk factors of interest were overweight/obesity, daily added sugar consumption, and sugar-sweetened beverage consumption. The protective factors of interest were physical activity and fruit and vegetable consumption (further described in methods).

##### Overweight/Obesity

Height and weight were objectively measured and used to calculate body mass index (BMI) as weight in kilograms divided by the square of height in meters. World Health Organization cut-points for overweight/obesity were age and sex specific for children and adolescents [18] and ≥25 kg/m^2^ for adults [19]. 

##### Diet

Dietary intake was measured via 24-h dietary recall using computer assisted interview instruments [20]. The Food Standards Australia New Zealand AUSNUT 2011–2013 food nutrients database was developed for this survey and enabled participants to report foods and beverages consumed using pictures or by identifying specific portions [21]. Fruit and vegetable consumption was based on the number of servings (vegetable serving = half a cup cooked vegetables, one medium potato or one cup of salad vegetables; fruit serving = one medium piece or two small pieces of fresh fruit, one cup of diced fruit) participants self-reported consuming on an average day. The Australian National Health and Medical Research Council recommendations were used to categorize participants who met and did not meet guidelines [22]. The proportion of added sugar in total daily energy consumption was calculated based on responses to the 24-h recall and the World Health Organization recommendations of less than 10% were used to categories those that met and exceeded daily added sugar intake [23]. Participants who reported having a sugar-sweetened beverage (defined as cordials, soft drinks, flavoured mineral waters, energy and electrolyte drinks, fortified waters, fruit and vegetable drinks that contain added sugar) at least once in the 24-h dietary recall were classified as consumers. 

##### Physical Activity

Physical activity was assessed via physical activity questionnaire asking participants items relating to their activity on each day in the last 7 days. For children and adolescents, items related to the type and time spent on activity transport, type and time spent on moderate to vigorous activity, and time spent on organized moderate to vigorous activity. Adults were asked, based on the same 7 days reference period, their time spent and frequency of walking moderate and vigorous physical activity, vigorous gardening, organized activity, strength and toning activities. Classification of child and adolescent physical activity was based on Australian National Physical Activity Recommendations, defined as at least 60 min of moderate to vigorous physical activity every day and no more than two hours of screen-based activity of entertainment and non-education purposes a day [24]. Adult physical activity guidelines of at least 150 min accumulated over a week period, across five sessions were used to classify emerging adults as sufficiently active, as consistent with national guidelines [24].

### 2.3. Statistical Analysis

All statistical analyses were conducted in Stata Corp. (College Station, TX, USA) and weighted to account for the multi-stage sampling technique and to reflect the wider Australian population. Significance was assumed at *p* < 0.05. Frequencies and weighted proportions were calculated and significance was calculated by logistic regression models across age groups, and for males and females within and across age groups. Logistic regression models were calculated separately for risk factors overweight/obesity, fruit and vegetable consumption, added sugar consumption, sugar-sweetened beverage consumption, and physical activity, with biological sex and the independent variable of interest. All models were adjusted for SEIFA index to account for socio-economic advantage/disadvantage.

### 2.4. Ethics Approval

Permission was obtained from the Australian Bureau of Statistics to access de-identified Basic Confidential Unit Record Files (CURFs), released on November 2014 to enable analysis for research purposes. Ethics approval for this study was granted from Alfred Health (Project No. 367/16).

## 3. Results

Participant characteristics are reported in Table 1 and regression models for sex differences for individual risk factors are reported in Table 2. 

### 3.1. Participant Characteristics

The mean age of participants was 7 years for children, 13 years for adolescents, and 22 years for emerging adults and the proportion of participants across different levels of disadvantage appeared evenly distributed. The prevalence of overweight/obesity by age group was 26% among children, 29% among adolescents and 35% among emerging adults. The proportion of emerging adults who were overweight/obese was significantly different compared to children and adolescents (*p* < 0.05). Overweight/obesity was significantly higher among emerging adult males (40%) than male children (24%) and male adolescents (31%). Close to half of the children (44%) met Australian dietary guidelines for daily fruit and vegetable consumption and this was significantly higher than adolescents (10%) and emerging adults (3%) (*p* < 0.05). The same significant trends (*p* < 0.05) were observed among male children (44%) compared to adolescent (8%) and emerging adult (2%) males, and female children (43%) compared to adolescent (13%) and emerging adult (4%) females. The proportion of individuals who met WHO guidelines for added sugar consumption was greater among emerging adults (45%) compared to adolescents (36%), and this was shown independently for emerging adult compared to adolescent males (47% vs. 38%) and emerging adult compared to adolescent females (44% vs. 33%). No other differences were observed. Adolescent males were the most frequent consumers of SSBs, with over half (55%) consuming on the day prior interview. The total proportion of SSB consumers was higher among adolescents (53%) compared to children (46%) (*p* < 0.05) and emerging adults (48%) (non-significant). 

### 3.2. Sex Differences Across Age Groups for Individual Risk Factors

Regression models revealed significant (*p* < 0.05) differences in risk factors for males compared to females. No significant differences emerged between male and female children. Among adolescents, females were almost twice as likely as males to meet fruit and vegetable consumption recommendations (OR = 1.84, 95% CI = 1.16, 2.93, *p* < 0.05). Prevalence of overweight/obesity was significantly lower among emerging adult females than males in the same age category (OR = 0.65, 95% CI = 0.44, 0.95, *p* < 0.05). Emerging adult females were less likely than males to have consumed a SSB on the previous day (OR = 0.61, 95% CI = 0.43, 0.86, *p* < 0.05), and were more likely to meet physical activity recommendations (OR = 1.44, 95% CI = 1.01, 2.06, *p* < 0.05). No other sex differences were observed. 

## 4. Discussion

Various sex-specific associations were found during adolescence and emerging adulthood, whereas male and female children (aged 5–9 years) tended to demonstrate similar risk and protective factor characteristics. Adolescent females were more likely than adolescent males to meet fruit and vegetable guidelines. Emerging adult females were less likely than emerging adult males to be overweight/obese, and to have consumed an SSB during the 24-h recall. The proportion of emerging adult females who met physical activity guidelines was significantly greater than emerging adult males. The sample was weighted to reflect the wider Australian population. Although health trends such as a quarter of children being overweight/obese, 3% of adolescents meeting physical recommendations and 3% of emerging adults meeting fruit and vegetable guidelines were consistent with previous estimates, these findings are nonetheless concerning. 

The prevalence of childhood overweight/obesity in this study mirrored that of previous national and international estimates [26]. While close to half of the child group met fruit and vegetable guidelines, there were also over half who exceeded added sugar recommendations and who consumed an SSB during the 24-h recall. The consumption of excess added sugar is of particular concern as evidence suggests that increasing added sugar consumption is paralleled by decreased nutrient and food group intakes [27]. Additionally, SSB consumption during childhood is associated with a range of health outcomes including overweight/obesity [28], metabolic risk [29], and dental caries [30]. While a greater proportion of children met physical activity recommendations compared to adolescents, this proportion was far smaller than that among emerging adults, and this holds important implications for parents, schools and community organizations that support and enable child physical activity. It is also noted that the difference in proportions meeting physical activity guidelines is likely to be explained at least in part by a target in adolescence that could be considered less attainable (60 min per day) compared to adulthood (150 min per week). No sex differences emerged in regression analyses suggesting male and female behavioural trends were similar during childhood.

While overweight/obesity prevalence was similar among children and adolescents, there were a significantly lower proportion of adolescents meeting fruit and vegetable and physical activity recommendations compared to children. Furthermore, a greater proportion of adolescents consumed an SSB in the 24-h recall compared to children. Previous research has demonstrated adolescence as a heterogeneous life phase characterized by significant physical and emotional development that impacts upon health risk behaviours [31]. Poorer health behavioural outcomes during adolescence compared to childhood, such as decreases in physical activity, have been previously reported [32]. As young people enter adolescence, they are more likely to make independent health choices, and are allowed greater autonomy that was previously governed by parents. Research has also shown that risk behaviours during adolescence are likely to cluster as multiple risk behaviours and, without intervention, persist into adulthood [33]. These current findings demonstrate the need for ongoing public health research, advocacy and policies specific to the adolescent age group to tackle population chronic disease. 

Adolescent females were significantly more likely than adolescent males to meet fruit and vegetable recommendations, however, the proportion overall was very low (90% did not meet recommendations). Although consistent with previous epidemiological evidence [34], this finding is particularly concerning with higher consumption of fruit and vegetables significantly associated with reduced risk of cancer [35], coronary heart disease [36], and other chronic diseases. It is also evident that adolescent males and females were equally as likely to exceed added sugar recommendations, consume an SSB, and fail to meet physical activity guidelines, which is inconsistent with previous evidence to date. Previous research suggests adolescent males typically consume SSBs and foods comprising high levels of added sugars at higher rates than adolescent females [37]. Physical activity participation among females has been shown to decrease in adolescence, compared to steady participation among males [32]. No such sex difference was observed in physical activity patterns among male and female adolescents in this study, suggesting this group are universally likely to benefit from physical activity promotion efforts. While health behavioural changes are expected to occur with increasing autonomy and developmental changes during adolescence, these current findings suggest that health promotion efforts can broadly target young people irrespective of sex, and are urgently needed particularly to increase fruit and vegetable consumption and encourage physical activity. 

In the current study, emerging adults had a cluster of lifestyle risk factors, such as low fruit and vegetable consumption, a high proportion consuming SSBs and having overweight/obesity, which are associated with adverse health outcomes in adulthood. Previous research has highlighted that emerging adults are at increased risk for excess weight gain [11]. Physical activity has also been shown to decrease during this stage [38], although this was not supported by the current study. There is evidence to suggest that lifestyle behaviours and weight status strongly track over time, highlighting the importance of establishing healthy behaviours at an early life stage [39]. As emerging adults are likely to have greater agency and ability to make decisions for themselves, this is a key opportunity to intervene regarding nutrition education, cooking skills, the importance of physical activity, while tailoring strategies to some of the challenges of this life stage. Emerging adulthood is a critical period of transition, and while this phase is marked by relative employment, residential and relationship instability, it remains a key time for establishing identity and self-efficacy [40]. Certainly, emerging adults have greater independence and often capacity for self-reflection, and thus warrant consideration as a unique developmental period from a public health/prevention perspective [41]. 

In this study, certain risk factors were poorer in males including weight status, dietary factors and physical activity. Interestingly, this is contrary to previous evidence suggesting physical activity declines in females at this age [42]. Previous literature has shown gender and sex differences in various aspects of health, such as higher quality diet among females, although poorer overall self-rated health [43]. There is evidence indicating that men in this age group show less interest in nutrition advice or health enhancing behaviours [44], and these differences have been suggested to reflect gender-based sociocultural expectations (e.g., messages surrounding female body composition/image, traditional roles surrounding food preparation). Given the lifestyle differences that emerge in this life stage and the potential for these differences to track through to adulthood, public health strategies may benefit from consideration of social and cultural factors associated with health behaviours among men and women. 

### Strengths and Limitations

While the cross-sectional nature of this study does not allow for the tracking of lifestyle behaviours over time, this study describes the lifestyle risk factors among a representative sample of both urban and rural Australians across various phases of early life. Importantly, the current study is strengthened by its examination of multiple lifestyle risk factors, including diet, physical activity and weight status, in order to more comprehensively capture lifestyle-related risk. Various lifestyle measures (dietary intakes, physical activity) were self-reported, and are thus susceptible to report bias, particularly among parents who may experience social pressure surrounding health behaviours of their children [45]. Although efforts were made to minimise bias in the NNPAS, previous research suggests there may be sex differences in levels of report bias, whereby females are more likely to misreport due to social desirability factors (e.g., dieting behaviours, perceived healthfulness) [46,47,48]. Further, by examining childhood through to emerging adulthood, this study is likely to capture the transition from parent-guided to self-guided health behaviours. Finally, the examination of lifestyle factors by sex provides information on when lifestyle risk factors may diverge, and may highlight particular windows of opportunity where intervention is likely to be impactful, or where targeted, sex-specific intervention strategies may be beneficial. 

## 5. Conclusions

While the underlying mechanisms are likely to span biological, sociological, environmental and other domains, the period of adolescence and emerging adulthood appear critical in the development of sex-specific differences in health behavioural trends. These findings are expected to hold critical implications for risk factor surveillance and the development of prevention efforts targeting diet, physical activity and other modifiable factors, as the engagement in these behaviours appears different for males and females as well as across age groups. While females overall tended to demonstrate behaviours considered more healthful compared to males, the prevalence of overweight/obesity, low adherence to fruit and vegetable, added sugar and physical activity guidelines, was concerning. This further rationalizes current prevention efforts to curb unhealthy weight gain through approaches that consider the complex environments in which behaviours occur involving individual, community and other contributing factors. Sex-specific differences among children were not observed in this study and this supports universal prevention programs for this age group, given males and females do not yet appear to be demonstrating unique health behaviours. Future research should aim to build on the largely self-reported descriptors utilised in this study, to consider the biomedical, environmental and socio-cultural experiences of individuals within these age groups. 

## Figures and Tables

**Table 1:** Demographic and health profile of children (5–9 years, *n* = 739), adolescents (10–17 years, *n* = 1304) and emerging adults (18–25 years, *n* = 909) in Australia, percentages weighted to reflect wider Australian population.

Characteristic	Children (5–9 Years)			Adolescents (10–17 Years)			Emerging Adults (18–25 Years)		
	Males, *n* = 368 (50.3)	Females, *n* = 371 (49.7)	Total, *n* = 739 (22.6)	Males, *n* = 675 (51.9)	Females, *n* = 629 (48.1)	Total, *n* = 1304 (36.0)	Males, *n* = 430 (49.9)	Females, *n* = 479 (50.1)	Total, *n* = 909 (41.4)
Mean age, years (SD)	7.1 (1.4)	7.2 (1.5)	7.1 (1.4)	13.4 (2.3)	13.4 (2.2)	13.4 (2.2)	21.5 (2.2)	21.6 (2.3)	21.6 (2.3)
SEIFA index ^1^									
Lowest 20%	58 (16.0)	65 (17.7)	123 (16.9)	117 (19.4)	100 (14.3)	217 (17.0)	75 (18.4)	111 (22.9)	186 (20.7)
Second quintile	70 (20.7)	63 (15.6)	133 (18.2)	120 (19.4)	114 (18.8)	234 (19.1)	85 (17.9)	93 (17.5)	178 (17.7)
Third quintile	72 (18.7)	69 (17.8)	141 (18.2)	136 (19.5)	143 (24.4)	279 (21.9)	86 (18.8)	86 (21.3)	172 (20.0)
Fourth quintile	73 (16.5)	78 (20.0)	151 (18.2)	117 (18.3)	115 (19.5)	232 (18.9)	78 (17.8)	67 (14.5)	145 (16.2)
Highest 20%	95 (28.1)	96 (28.9)	191 (28.5)	185 (23.3)	157 (23.0)	342 (23.2)	106 (27.0)	122 (23.8)	228 (25.4)
Overweight/obesity *n* (%) ^2^	66 (24.1)	76 (27.5)	142 (25.8)	174 (30.7)	156 (27.4)	330 (29.2)	171 (39.8) ^a,b^	145 (30.1)	316 (35.1) ^c,d^
Fruit and vegetable *n* (%) ^3^	155 (44.4)	165 (42.7)	320 (43.6)	63 (7.5) ^a^	76 (12.9) ^a^	139 (10.1) ^c^	7 (2.0) ^a,b^	20 (4.0) ^a,b^	27 (3.0) ^c,d^
Free sugars *n* (% who met) ^4^	152 (39.9)	160 (41.0)	312 (40.4)	251 (38.4)	202 (33.3)	453 (36.0)	190 (46.5) ^b^	202 (44.2) ^b^	392 (45.3) ^d^
Sugar-sweetened beverage on day prior *n* (% yes) ^5^	162 (46.0)	160 (46.1)	322 (46.1)	379 (55.2) ^a^	328 (49.9)	707 (52.7) ^c^	241 (54.0) ^a,b^	206 (41.8) ^a^	447 (47.9)
Physical Activity *n* (% who met) ^6^	70 (17.4)	68 (14.3)	138 (15.8)	22 (3.3) ^a^	23 (3.4) ^a^	45 (3.3) ^c^	176 (42.3) ^a,b^	247 (52.1) ^a,b^	423 (47.2) ^c,d^

Note: *n*-values are raw sample size calculations and proportions are weighted to reflect wider Australian population. Significance is assumed at *p* < 0.05. Percentages sum vertically within age groups for SEIFA. SD, standard deviation; SEIFA, Socio-Economic Indexes for Areas. ^1^ SEIFA classified as per Australian Bureau of Statistics [17]. ^2^ Overweight/obesity defined by World Health Organisation [19], and age- and sex-specific cut-points equivalent to adult body mass index >25 kg/m^2^ for children and adolescents [18]. ^3^ Proportion who met fruit and vegetable Australian Dietary Guidelines [25] (4–7 years = 2 servings of vegetables, 1 serving of fruit; 8–11 years = 3 servings of vegetables and 1 serving of fruit; 12–17 years = 4 servings of vegetables, 3 servings of fruit; 18 years and over = 5 servings of vegetables and 2 servings of fruit). ^4^ Proportion who met World Health Organization recommendations of free sugars comprising less than 10% of total daily energy intake. ^5^ Reported consuming a sugar-sweetened beverage on day prior via 24-h recall. ^6^ Proportion who met Australian physical activity guidelines [24]. ^a^ Significantly different to children (within sex). ^b^ Significantly different to adolescents (within sex). ^c^ Significantly different to children (total). ^d^ Significantly different to adolescents (total).

**Table 2:** Odd Ratios (OR) for health characteristics (dependent variable) the role of sex (independent variable, male = 0, female = 1) in health trends for children, adolescents and emerging adults, weighted to reflect the wider Australian population and adjusted for Socio-Economic Index for Areas.

Dependent Variables	Children (5–9 Years)			Adolescents (10–17 Years)			Emerging Adults (18–25 Years)		
	OR	95% CI	*p*	OR	95% CI	*p*	OR	95% CI	*p*
Overweight/obese ^1^	1.22	0.74, 2.00	0.427	0.87	0.61, 1.23	0.418	**0.65**	**0.44, 0.95**	**0.025**
Fruit and vegetable consumption ^2^	0.94	0.64, 1.37	0.732	**1.84**	**1.16, 2.93**	**0.010**	2.05	0.61, 6.88	0.500
% energy from added sugars ^2^	0.95	0.65, 1.39	0.788	1.24	0.91, 1.68	0.169	1.09	0.77, 1.55	0.622
Sugar-sweetened beverages ^3^	1.01	0.69, 1.47	0.968	0.81	0.61, 1.10	0.168	**0.61**	**0.43, 0.86**	**0.005**
Physical Activity ^2^	0.79	0.48, 1.30	0.351	0.99	0.47, 2.07	0.983	**1.44**	**1.01, 2.06**	**0.043**

OR, odds ratio; 95% CI, 95% confidence interval; bolding indicates significance at *p* < 0.05. ^1^ Overweight/obese (=1) compared to normal weight (=0). ^2^ Met recommendations (=1) compared to those that did not met recommendations (=0). ^3^ Consumers (=1) compared to non-consumers (=0). OR relates to likelihood of females compared to males for each specific health risk factor (e.g., the odds ratio for females being classified as overweight/obese compared to males among children was 1.22).
